# DDX41胚系突变与骨髓增生异常肿瘤

**DOI:** 10.3760/cma.j.cn121090-20251118-00537

**Published:** 2026-07

**Authors:** 玥 仲, 志坚 肖

**Affiliations:** 1 北京协和医学院，中国医学科学院血液病医院（中国医学科学院血液学研究所），血液与健康全国重点实验室，国家血液系统疾病临床医学研究中心，细胞生态海河实验室，天津 300020 State Key Laboratory of Experimental Hematology, National Clinical Research Center for Blood Diseases, Haihe Laboratory of Cell Ecosystem, Institute of Hematology & Blood Diseases Hospital, Chinese Academy of Medical Sciences & Peking Union Medical College, Tianjin 300020, China; 2 天津医学健康研究院，天津 301600 Tianjin Institutes of Health Science, Tianjin 301600, China

## Abstract

DDX41突变是骨髓增生异常肿瘤患者中最常见的胚系突变之一，伴DDX41胚系突变骨髓增生异常肿瘤患者在发病年龄、预后、致病机制及治疗反应方面均具有独特性。本文在概述其分子流行病学及临床特征的基础上，重点综述DDX41突变“二次打击”致病机制的研究进展，系统总结不同治疗策略的疗效及预后，并讨论了现有骨髓增生异常肿瘤预后评分系统在该类患者中的适用性及局限性，以期为患者的风险评估、临床诊治及全病程管理提供参考。

## Abstract (en)

DDX41 mutations represent one of the most frequent germline mutations in patients with myelodysplastic neoplasms, conferring unique clinical features of age of onset, prognosis, pathogenic mechanisms, and treatment response. This review summarizes the molecular epidemiology and clinical features of germline DDX41-mutated MDS, with a particular focus on recent advances in the “second-hit” model underlying disease pathogenesis. We further review the efficacy of current therapeutic strategies and discuss the applicability and limitations of existing prognostic scoring systems for this unique patient population. This review aims to provide an updated overview of current evidence and to inform risk stratification, clinical management, and long-term care of patients with germline DDX41-mutated MDS.

2016年版世界卫生组织（WHO）造血与淋巴组织肿瘤分类修订版正式将“具有胚系易感性的髓系肿瘤”作为独立疾病类别[1]，其中，DDX41突变是骨髓增生异常肿瘤（MDS）患者最常见的胚系突变之一。伴DDX41胚系突变MDS患者的发病年龄较晚，主要见于老年群体，且其临床特征、疾病预后及治疗反应等方面均具有独特性[2]–[5]，为了加深我国血液学工作者对伴DDX41胚系突变MDS的认识，规范对这类患者的临床诊治和全病程管理，本综述就该领域认识现况做一系统介绍。

一、分子流行病学

DDX41基因位于5号染色体长臂（5q35.3），编码一种DEAD-BOX ATP依赖性RNA解旋酶，参与RNA转录、剪接、核糖体生物合成及翻译等多种关键细胞过程。DDX41蛋白主要分为4个结构域：N端结构域、DEAD盒结构域、解螺旋酶C结构域和C端结构域[6]。

DDX41突变是MDS患者中最常见的胚系突变之一，其携带率和致病风险在不同人群中有所差异[3]。在普通人群中，DDX41胚系突变的携带率约为0.78％，其中致病性突变携带率为0.23％[7]，男、女突变携带率相似。在普通人群队列中，DDX41致病性胚系突变携带者在约13年的随访期间发生MDS或急性髓系白血病（AML）的累积风险约为3.21％，男性携带者进展为髓系肿瘤的风险更高[3],[7]–[11]。在MDS患者中，DDX41致病性或可能致病性胚系突变的携带率显著增高，约4.62％[3]。与许多其他早发性胚系易感髓系肿瘤不同，携带DDX41胚系突变的MDS患者发病年龄通常较晚，集中在60～70岁，与散发型MDS的发病年龄相近[7],[11]。此外，DDX41胚系突变的分布存在地区差异，亚洲人群的携带率高于欧洲人群[3]。具体而言，亚洲人群中最常见的胚系突变为p.A500fs，而欧洲人群则以p.D140fs、p.M1I为主要突变类型[3],[7]–[8],[12]。虽然DDX41胚系突变的分布有地区差异，但MDS患者携带的胚系突变几乎均为截短突变[3]。与之不同的是，DDX41体细胞突变类型并未表现出明显的地域分布特征，且绝大多数属于非截短突变，其最主要的突变类型为p.R525H。此外，目前尚未在患者中检测到双等位基因截短突变，提示纯合的截短突变可能导致胚胎致死。

DDX41胚系突变与体细胞突变之间存在密切关联。大多数携带DDX41胚系突变MDS患者同时伴有DDX41体细胞突变，其中最主要的体细胞突变位点为p.R525H[2],[13]–[14]。而在无胚系突变的背景下，MDS患者中出现孤立DDX41体细胞突变的比例极低，仅为0.4％[13]。这一现象提示：①DDX41体细胞突变很少单独作为致病因素；②它很可能是DDX41胚系突变致病机制中的关键环节。在伴有DDX41胚系突变的MDS患者中，其获得性体细胞p.R525H突变的变异等位基因频率（VAF）中位数约10％[4],[15]。

除DDX41体细胞突变以外，伴DDX41胚系突变的患者多数还会携带其他体细胞突变，其中常见的突变基因有ASXL1、DNMT3A、TET2和TP53[3],[8],[12],[16]–[17]。值得注意的是CUX1突变与疾病进展的关联。在原始细胞比例较高的MDS患者中，携带DDX41胚系突变的个体比不携带该突变的野生型个体更容易出现CUX1共突变[3]；而在原始细胞比例较低的患者中，这一差异并不显著。这种现象提示，在DDX41胚系突变的基础上，伴CUX1突变可能与疾病进展及原始细胞增殖有关。

二、DDX41突变导致MDS的分子发病机制

DDX41蛋白作为DEAD-box RNA解旋酶家族成员，在维持基因组稳定性、协调RNA剪接及转录延伸等过程中发挥核心作用，其功能缺失可导致R-loop与G-四链体（G4）异常积聚，进而诱发DNA损伤与复制压力[18]–[21]。

已有研究结果表明DDX41突变的致病机制符合“二次打击模型”。DDX41^–/–^小鼠均出现了急性骨髓衰竭并快速死亡；而与其相反，DDX41^+/–^小鼠在较长时间仍可维持近乎正常的造血功能，这说明单个功能性DDX41等位基因足以支持一段时间的稳态造血[15],[20]。其中，截短突变常作为“肇始性打击（first hit）”：接受DDX41^+/–^骨髓细胞移植的受体鼠虽然生存期与野生型无明显差异，但在15月龄时出现了骨髓增生减低和髓系发育异常表型[15],[17]，这与临床观察到的伴胚系DDX41截短突变MDS患者发病年龄较晚（60～70岁）相吻合。而后天获得的体细胞突变将进一步削弱DDX41功能，促进疾病转化。如p.R525H突变作为一种亚功能突变，加剧了肇始性打击引起的R-loop、G4积聚以及DNA损伤[18],[22]–[23]。即使是VAF<20％的p.R525H突变，也足以在小鼠模型中引发显著血细胞减少及髓系发育异常[15]，说明二次打击即使克隆负荷较低，仍对疾病演变有重要影响。

DDX41突变所致骨髓衰竭主要源于其在红系分化早期阶段的关键作用。研究结果显示，EpoRCre:Ddx41^fl/+^小鼠在2月龄时出现轻度的大红细胞性贫血，提示DDX41杂合缺失会损害红系稳态[20]。进一步研究发现，在红系祖细胞阶段纯合性敲除DDX41（EpoRCre:Ddx41^fl/fl^），会导致胚胎致死，这表示DDX41对早期红系造血至关重要[20]。而在晚期阶段纯合性敲除DDX41（HBBCre:Ddx41^fl/fl^），则对小鼠生存期及血象均无显著影响[20]。这一阶段特异性致死表型与DDX41的G4解旋功能密切相关。正常情况下，DDX41可直接结合并解旋G4结构；若其功能下降时，G4积聚会引发红系基因组不稳定、核糖体生物合成缺陷、p53信号上调以及cGAS-STING通路的过度激活[20]。尽管p53信号被激活，但p53基因缺失并不能挽救造血系统特异性DDX41基因敲除所致的胚胎致死表型。相反，cGAS缺失可完全挽救该表型，表明cGAS-STING通路是DDX41缺失导致红系衰竭的关键机制[20]。此外，有研究显示，DDX41表达量降低时，细胞内R-loop会异常积聚，最终影响DNA的复制和转录[18],[24]。而G4积聚和R-loop形成之间存在正向反馈循环，进一步加剧红系分化受阻的程度[25]。此外，在来自MDS患者骨髓的红系集落形成单位中，同时携带DDX41胚系突变及p.R525H突变的细胞表现出G_2_/M期细胞周期停滞、R-loop积聚和DNA损伤[22]。这一现象表明，后天获得的p.R525H突变可在胚系突变的基础上进一步损害红系生成。

目前对DDX41突变驱动向髓系肿瘤转化的分子机制研究仍较少。已有研究表明，除了DDX41自身发生二次体细胞突变外，TP53的杂合缺失也是常见的协同事件[26]。接受DDX41^+/–^；Trp53^+/–^骨髓细胞移植的受体鼠出现血液系统恶性肿瘤比例明显升高，其表型类似人类MDS/AML，表现为贫血、血小板减少、干细胞扩增和髓外造血等特征[17]。这提示DDX41突变所致髓系肿瘤恶性转化可能需要其他基因突变的协同作用，而TP53突变可能在其中发挥关键作用。此外，有研究表明：除了细胞自主性缺陷，DDX41突变还可能通过非自主方式影响骨髓微环境，例如p.R525H突变细胞能够调控分泌细胞因子（TGF-β1、ICAM-4等）影响其他正常细胞[22]，可能是驱动恶性转化的机制之一。

三、伴DDX41胚系突变MDS患者的临床和实验室特征

多数患者在确诊前存在持续的血细胞减少史，通常超过1年[27]。其中贫血尤为常见。研究表明，在p.D140fs携带者中，可观察到轻度的红细胞减少[7]。关于白细胞计数的研究结果存在差异：部分研究报道该类患者白细胞减少较明显[3],[27]，但近期一项纳入20项研究的Meta分析显示，DDX41突变与白细胞减少之间没有明确关联[26]。骨髓细胞形态学方面，此类患者骨髓增生程度减低，但骨髓原始细胞比例较高[2],[11],[27]–[28]。在细胞遗传学方面，该类患者大多数为正常核型[11],[27]，而异常核型者则以复杂核型多见[26]。

四、伴DDX41突变MDS患者的治疗

有研究指出，鉴于携带DDX41突变的MDS病程相对惰性，对于血液学指标尚可的患者，即使骨髓原始细胞比例较高，也可先观察随访，待血液学指标明显变化再开启治疗[29]–[30]。

已有多项小系列研究报道此类患者对去甲基化药物治疗反应良好[3]–[4]，伴DDX41突变的患者在接受阿扎胞苷（75 mg·m^−2^·d^−1^×7 d，每28 d为1个周期）治疗后，其总生存（OS）期显著延长，且DDX41基因突变是OS的独立保护因素[4]。我们的回顾性研究亦得出了相似的结论：在6例接受阿扎胞苷联合维奈克拉作为初始治疗的患者中，有5例达到了完全缓解（CR）[31]。如果去甲基化治疗失败，改用去甲基化药物联合维奈克拉方案可能有效，Nanaa等[32]报道3例携带有DDX41致病性突变的MDS患者在去甲基化治疗失败后，联合使用去甲基化药物和维奈克拉治疗，均达到CR。

来那度胺是一个可考虑的替代选择（10 mg/d×21 d，每28 d为1个疗程）[33]。研究表明，对于非5q–综合征患者，约25％的患者对来那度胺敏感，而DDX41突变与更高的治疗反应率有关[14],[34]–[35]。我们之前曾报道过一例正常核型、伴有DDX41c.572-1G>C和p.P379Q突变的MDS-EB2患者在来那度胺单药治疗2个疗程后即获得血液学改善[36]。最近我们的回顾性研究显示，在接受来那度胺单药治疗的20例携带DDX41突变的MDS患者中，总缓解率达到75％，其中CR 4例，骨髓完全缓解（mCR）1例，骨髓完全缓解伴血液学改善（mCR+HI）2例，血液学改善（HI）8例，OS期达49.5个月[37]。进一步分析发现，治疗有效与DDX41体细胞突变VAF下降相关；而在疾病进展或治疗失败的患者中，多数出现了TP53的克隆演变，提示其可能与来那度胺耐药有关[37]。

Nyquist等[38]报道既往去甲基化治疗无效或不能耐受的10例携带DDX41突变合并骨髓低增生的患者，采用低剂量（2 mg/d）马法兰，治疗持续至获得最佳疗效（通常为8~12周）停药，随后进入治疗间歇期，期间不予任何抗肿瘤药物治疗，当出现疾病复发的证据时，则开启新的治疗周期。结果显示，所有携带DDX41突变的患者均对低剂量马法兰有反应（总缓解率100％，其中mCR 6例，HI 4例），其OS期达25个月。

异基因造血干细胞移植（allo-HSCT）是MDS唯一的治愈性干预措施，然而不同的基因突变影响了MDS移植后的生存结局[39]。DDX41胚系突变相关患者发病年龄较晚，留给移植的窗口期较短。此外，在选择亲缘供者进行移植时，应谨慎排查其是否携带DDX41胚系突变。已有病例报道，亲缘供者携带DDX41胚系突变，患者在接受移植后第4年发生供者来源白血病[40]，因此对亲缘供者进行全面的基因筛查尤为重要。除了供者选择要格外注意之外，伴DDX41突变MDS患者是否优先考虑接受allo-HSCT尚存争议。有研究显示伴DDX41突变MDS患者移植后非复发死亡率较高[41]。另外，也有研究显示伴DDX41突变AML患者移植后无复发生存期更长，但并未转化成更长的OS[10],[30],[42]。关于移植后非复发死亡率较高，OS未明显延长的原因，Saygin等[43]认为可能与DDX41胚系致病性突变患者严重的移植物抗宿主病（GVHD）发生率较高有关，而移植后应用环磷酰胺（50 mg·kg^−1^·d^−1^，+3、+4 d，静脉给药）可以有效降低GVHD的发生率[44]。

五、伴DDX41突变MDS患者的预后

目前常用的MDS预后评分系统，如修订版国际预后积分系统（IPSS-R）和分子国际预后积分系统（IPSS-M），在预测携带DDX41突变患者的预后方面存在局限性[3],[45]。研究显示，虽然伴有DDX41致病性胚系突变的患者，其IPSS-R评分多属于中危或高危[3],[11],[42]，较DDX41野生型患者评分更高，但这类患者的实际预后反而更好[2]–[4],[8],[30]。进一步分析发现，DDX41截短型突变是患者进展为AML的独立危险因素[3]，然而，无论是DDX41截短型突变，还是TP53突变、SRSF2突变、异常核型、原始细胞比例等传统MDS预后预测因子，均未能有效预测该类患者的整体预后[8]。此外，尽管IPSS-M在该人群中的预测能力较为有限，但当研究者将患者类别简化为“极高危+高危”以及“其他”两组时，前者OS期显著较短[45]。同时，研究显示PLT>150 ×10^9^/L与较长的OS期相关[45]。因此，伴DDX41突变MDS患者预后评估仍颇具挑战性。此外，对DDX41胚系突变携带者的髓系肿瘤演变也缺乏有效的风险分层。目前已有研究表明，获得性DDX41体细胞突变的出现及平均红细胞体积升高可能预示从携带者阶段向MDS进展的风险增加[7],[46]。

六、伴DDX41突变MDS患者的遗传咨询与随访建议

根据《北欧成人髓系肿瘤胚系易感诊疗指南》，当体细胞NGS检测显示DDX41基因突变占比处于40％~60％时，提示该变异可能为胚系起源。在此情况下，应建议患者利用造血系统外组织进行胚系验证。若该DDX41突变类型为截短突变，则进一步支持其为胚系来源的可能性[5]。

对于确诊携带胚系DDX41易感突变的患者，应为其本人及家属提供全面的遗传咨询，以识别具有携带风险的亲属。患者的一级亲属携带DDX41胚系突变的概率为50％，在患者需要进行allo-HSCT的情况下，遗传咨询能够帮助识别并排除携带DDX41突变的亲属作为移植供者，从而降低供者来源白血病的风险。此外，由于携带DDX41突变MDS具有晚发性特点，完善遗传咨询有助于对无症状DDX41突变携带者进行明确诊断。这有利于对携带者建立定期血液学监测，从而实现对疾病进展的早期诊断和早期治疗。对于有生育计划的患者，还应开展备孕前遗传咨询。

所有携带胚系DDX41易感突变的个体，无论其当前临床表现如何，均应在血液专科接受系统随访。根据指南建议，确诊初期应完成以下基线评估：血常规、骨髓穿刺/活检、骨髓组织NGS测序以及其他器官的系统性检查。后续随访方案为每半年复查血常规，每年复查外周血NGS，若血常规有明显变化，则需及时复查骨髓穿刺[5]。

七、结语

综上所述，DDX41胚系易感MDS是一个由“二次打击”驱动，具有晚发、男性优势及惰性病程等特点的疾病亚型。尽管此类患者的预后较好，但传统的预后分层系统对其预后评估价值有限。治疗方面，此类患者对去甲基化药物等反应良好。未来仍需开展更多针对预后评估及治疗的研究以促进携带DDX41突变MDS患者的早期识别、治疗及预后评估。在我国，首先应重视胚系易感髓系肿瘤的临床识别和诊断，建立这类患者的全病程管理体系；其次，利用我国的病例资源优势，建立全国性病例队列和数据库并开展前瞻性、多中心临床试验研究，在国际上发出中国声音，贡献中国方案。
